# A Case-Control Study of Meat Mutagens and Colorectal Cancers in Viet Nam

**DOI:** 10.31557/APJCP.2020.21.8.2217

**Published:** 2020-08

**Authors:** Hang Viet Dao, Tu Thi Minh Nguyen, Hoc Hieu Tran, Linh Thuy Dang, Minh Thi Dinh, Ngoan Tran Le

**Affiliations:** 1 *Hanoi Medical University, Hanoi, Viet Nam. *; 2 *School of Biotechnology and Food Technology, Hanoi University of Science and Technology, Hanoi, Viet Nam. *; 3 *Institute of Research and Development, Duy Tan University, Da Nang 550000, Viet Nam. *; 4 *Department of Public Health, School of Medicine, International University of Health and Welfare, Japan. *

**Keywords:** Colorectal cancer, meat mutagens, cooking method, beef intake

## Abstract

**Objective:**

The aim was to examine the association between heterocyclic amines 2-amino-1-methyl-6-phenylimidazo pyridine (PhIP) and the risk of colorectal cancer (CRC) in Viet Nam.

**Methods:**

We performed a case-control study for 512 colorectal cancer patients with the histopathological confirmation and 1,096 hospital controls. We collected information on lifestyle, diet, and cooking methods from participants by trained interviewers using the validated questionnaires. We used data of PhIP concentration in cooked beef analyzed by the LC/MS/MS and cooking questionnaire to estimate the daily intake of PhIP. We divided the estimated amount of PhIP (ng/person/day) into three levels of non-intake (the reference), medium, and high to estimate the Odds ratio and 95% confidence interval (OR, 95%CI).

**Results:**

The median intake of PhIP (ng/person/day) was 18ng and 102.8ng for medium and high PhIP intake, respectively. There was a significant association between PhIP intake and the risk of colorectal cancer. The adjusted OR (95%C), high intake vs. non-intake, were 4.89 (3.03, 7.89), *p_trend*<0.01 for all participants, for men OR (95%C): 5.27 (2.83, 9.81), *p_trend*<0.01, for women OR (95%C): 4.58 (2.10, 10.01), *p_trend*<0.01. The significant positive association was also observed for the sub-sites of the colon (proximal and distant) and rectum cancers.

**Conclusions:**

We observed the positive association of PhIP contributed from cooked-beef and the development of CRC. Cooking methods related to the occurrence of PhIP and other types of heterocyclic amines in cooked-beef and other meats would be significant investigations.

## Introduction

A rapid significant increase of both colorectal cancer (CRC) mortality and meat intake has been seen at populations in Viet Nam from 1981 to date. The age-standardized mortality rate per 100,000 (Segi-standard world population) in the Nghe An province continuously increased from 2.65 (in 2005-2006) to 2.80 (2007-2008), 3.30 (2009-2010), 4.24 (2011-2012), and 4.36 (2013-2014) (Thuong, 2018). Meat consumption per person per day has steadily increased from 11.1g (1981-1985) to 24.4g (1987-1989), 51.0g (2000), and 84.0g (2010); pork and beef are the main red meat intake in Viet Nam (Ministry of Health and National Institute of Nutrition, 2010). While the consumption of red meat is probably carcinogenic to humans and the target organs were colorectal, pancreases, and prostate (Group 2A) (IARC, 2018), the association of meat intake and the occurrence of colorectal cancer in the country and Asia region is still limited. The underlying etiology of red meat induced cancer might be dietary chemical carcinogens generated in cooked meat at high cooking temperatures. There were over 20 types of heterocyclic amines contaminated in our cooked meat. Major HCAs found in human diet include 2-amino-3,8-dimethylimidazo quinoxaline (MeIQx), 2-amino-1-methyl-6-phenylimidazo pyridine (PhIP), 2-amino-3,4,8-trimethylimidazo (DiMeIQx). Animal studies have provided sufficient evidence for the carcinogenic potential (Group 2B-IARC) of MeIQx, DiMeIQx and PhIP (IARC, 1993; NTP, 2014; Sugimura, 1997).

Heterocyclic amines 2-amino-1-methyl-6-phenylimidazo pyridine (PhIP) has detected in steak (1.9 to 30 ng/g) but was formed only in very well done fried or grilled hamburger (Sinha et al., 1998). Animal studies have provided sufficient evidence for their carcinogenic potential (Group 2B, International Agency for Research on Cancer, IARC) of PhIP (IARC, 1993; NTP, 2014). 

The aim was to examine the association between heterocyclic amines 2-amino-1-methyl-6-phenylimidazo pyridine and the risk of colorectal cancer in the North Viet Nam.

## Materials and Methods 

*Study population*

A case-control study performed for 512 colorectal cancers and 1096 hospital controls. The study participants recruited from the in-patient departments of three University Hospitals in the Hanoi city from Dec. 2017 to Dec. 2018.

*Case ascertainment*

**Figure 1:**
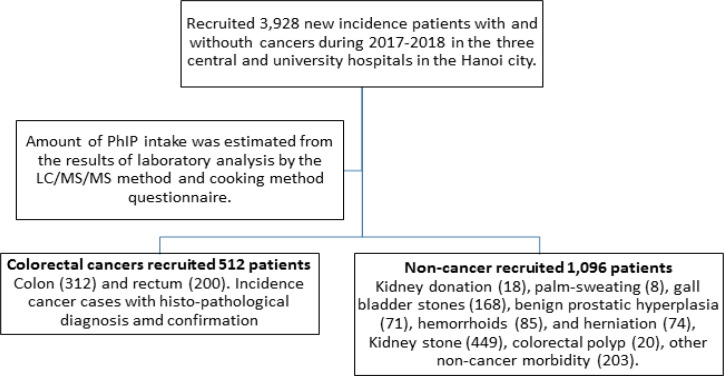
Flow Chart of the Recruited Study Participants

Among a total of 3,928 recruited incidence patients, we identified 512 cases diagnosed colorectal cancer by histopathological confirmation, Figure 1. Colorectal cancers included 312 colon cancer cases and 200 rectum cancer cases. Among colon cancer, it was 102 proximal colon, 93 distal colon, and another 117 cases. Among total colorectal cancer, men were 292, and women were 220 cases.

*Control recruitment*

Controls were 1096 non-cancer patients: Kidney donation (18), palm-sweating (8), gall bladder stones (168), benign prostatic hyperplasia (71), hemorrhoids (85), and herniation (74), Kidney stone (449), colorectal polyp (20), other non-cancer morbidities (203). They admitted to the same in-patient departments for weekly surgery. They were cancer-free on the recruited time and in their lifetime health history. 

*Exclusion*

We excluded both cancer cases and controls in case they were unable to communicate due to advanced disease stages; patients having substantial changes in their diet due to metabolic disorders, and diabetes; and patients refused to participate in the study.

*Assessment of diet and cooking methods*

We used the validated semi-quantitative food frequency questionnaire to collect the dietary history of participants in the past year, before the onset of cancer or non-cancer diseases. The SQFFQ has good characteristics of feasibility, practical, and reliability in the general population. The SQFFQ included 97 foods/recipes of 12 groups of foods that included cereals (9 items), peanuts and beans (7) vegetables (24), fruits (10), fat and oil (3), red and white meats (22), fishes (10), eggs (4), milk and its products (3), snacks (2), salt and fish sauce (2), and alcohol or vodka (1). We classified the food frequencies of intake into seven categories: never or seldom, 6-11 times/year, 1-3 times/month, 1-2 times/week, 3-4 times/week, 5-6 times/week, and 1-3 times/day (Le et al., 2018).

For tobacco smoking, we classified all participants into three categories: never smokers, ex-smokers, and current smokers in their lifetime. Never smokers were those who never smoked completely only one cigarette or waterpipe tobacco (WPT) smoking in their lifetime. For smokers, the information on types of tobacco (cigarette, WPT, or both types), the average number of cigarette and WPT per day during their age of 15-20, 21-25, 26-30, 31-40, 41-50, 51-60, 61-70, and 71+ (if applicable), the duration of smoking (current smokers), and the duration of quit smoking (current and ex-smokers) were obtained. Finally, we divided participants into two groups of lifestyle never-smokers and ever smokers for adjustment in the multivariable-adjusted model (Le et al., 2018). 

For cooking methods, a questionnaire inquired about the frequency of intake (i.e. never or less than six times/year, 6-11/year; 1-3/month, 1-2/week, 3-4/week; 5-6/week, and 1-3/day). Also, doneness levels included four categories (i.e. lightly browned; medium browned; well browned, and blackened/charred) of cooked beef (i.e. pan-fried hamburger, pan-fried steak, roast beef, and barbecued steak). We have checked the accuracy of obtained data using this cooking questionnaire by the measurement of observer agreement for categorical data as a generalized kappa-type statistic, and estimation of sensitivity and specificity. We evaluated the repetition of cooking methods of beef in a sample of 292 participants. Each participant completed twice the cooking questionnaire one month apart during the intervening month. Participants consumed at least one of five types of cooking pan-fried hamburger; pan-fried steak, roast beef, and barbecued steak were counted as the eating group, code as 1, the other code as 2 for both the first and second completed the cooking questionnaire. The estimated Kappa (0.55) was good and the agreement as high as 86%. The estimated sensitivity (70.6%) and specificity (89.2%) was also very good (non-published data). Therefore, data on cooking methods of beef was reliable to estimate the amount of PhIP intake per day per person in the present study.

*Assessment of HCA intake*

The experience investigators examined PhIP concentration in cooked beef in the laboratories of the School of Biotechnology and Food Technology, Hanoi University of Science and Technology, and the Center for Research and Technology Transfer, Vietnam Academy of Science and Technology (Linh, 2019). They performed LC/MS/MS method to analyze PhIP and MeIQx in 14 fried and grilled food samples, represented for the regular meals of participants from three communities represented three provinces in the Red Delta River in the North Viet Nam. The estimated concentration (C, ng/g) of PhIP was 77.2 ng/g in the part of blackened/charred barbecued steak and 10.4 ng/g in the part of blackened/charred pan-fried steak, pan-fried hamburger, roast beef. We assumed that the PhIP concentration by doneness levels will be balanced (B, %) to be 100% in the part of blackened/charred, then it was 75% in the well browned, 50% in the medium browned, and 25% in the lightly browned of cooked-beef. 

The average amount (A, gram (g)) of intake of cooked beef recipes was 33.3g, 170.0g, 136.5g, and 66.7g for the pan-fried hamburger, pan-fried steak, roast beef, and barbecued steak, respectively (Linh, 2019). Based on the obtained information provided by participants of the frequency of intake (i.e. never or less than six times/year, 6-11/year; 1-3/month, 1-2/week, 3-4/week; 5-6/week, and 1-3/day), we counted a total frequency of intake (F) of beef serving times per year. The estimated amount of PhIP from the beef of intake calculated by the following formulation:

(A: The average amount (A, g) of intake of each cooked beef recipe; B: the balanced PhIP concentration by doneness levels (B, %); C: The estimated concentration (C, ng/g) of PhIP intake; F: Total frequency of intake (F) of beef serving times per year).

*Data handle and statistical analyses*

Body Mass Index (BMI) was calculated as (BMI= weight (kg) / height ((m)^2^) for adjustment. We divided the exposure to PhIP into three levels of non-intake, medium, and high. The non-beefeater participants were the reference, crude, and adjusted Odds ratio and 95% confidence interval (OR, 95%CI) were estimated. Model 1: Adjusted for age groups (0-29, 30-39, 40-49, 50-59, 60-69, ≥70 ages) for men and women; and age groups and sex for a total. Model 2: Multivariable adjusted for age groups (0-29, 30-39, 40-49, 50-59, 60-69, ≥70 ages) for men and women; and age groups and sex for total; BMI (18.5 to <23, 23 to <25, ≥25, <18.5 kg/m^2^); education level (Primary school or under, secondary school, high school, higher high school, unknown); Frequency of intake of vegetables (Water spinach, mustard greens, sauropus, Malabar nightshade, cabbage); and lifetime smoking (cigarette, WPT, or both) (yes/no).

*Ethics consideration*

The ethic certificate of approval by the Hanoi Medical University IRB for the present study was issued on Dec. 25th, 2018 and by the International University of Health and Welfare IRB, Japan on May 17^th^, 2019. We obtained written informed consent from all participants of the present study.

**Table 1:** PhIP from Beef Intake and Risk of Colorectal Cancers

	Control	Cancer	Total	PhIP intake (Median)	Adjusted ^b^ OR (95%CI)	*P_trend*	Multivariable adjusted ^c ^OR (95%CI)	*P_trend*
CRC^ a^								
Men and women	1,015	402	1,417	Non-intake	1.00 (reference)		1.00 (reference)	
	50	57	107	18	3.27 (2.17, 4.93)		3.06 (2.00, 4.66)	
	31	53	84	102.8	4.86 (3.03, 7.80)	<0.01	4.89 (3.03, 7.89)	<0.01
Total	1,096	512	1,608					
Men	593	231	824	Non-intake	1.00 (reference)		1.00 (reference)	
	31	29	60	18	2.66 (1.56, 4.56)		2.77 (1.57, 4.88)	
	18	32	50	87	4.96 (2.69, 9.11)	<0.01	5.27 (2.83, 9.81)	<0.01
Total	642	292	934					
Women	422	171	593	Non-intake	1.00 (reference)		1.00 (reference)	
	19	28	47	18	4.30 (2.25, 8.21)		3.70 (1.91, 7.15)	
	13	21	34	112.5	4.70 (2.21, 9.98)	<0.01	4.58 (2.10, 10.01)	<0.01
Total	454	220	674					
Colon								
Men and women	1,015	241	1,256	Non-intake	1.00 (reference)		1.00 (reference)	
	50	37	87	18	3.59 (2.25, 5.72)		3.28 (2.02, 5.31)	
	31	34	65	97.5	5.65 (3.31, 9.63)	<0.01	5.68 (3.31, 9.76)	<0.01
Total	1,096	312	1,408					
Men	593	139	732	Non-intake	1.00 (reference)		1.00 (reference)	
	31	19	50	18	2.98 (1.61, 5.52)		2.86 (1.48, 5.52)	
	18	23	41	76.5	6.47 (3.31, 12.67)	<0.01	6.67 (3.35, 13.3)	<0.01
Total	642	181	823					
Women	422	102	524	Non-intake	1.00 (reference)		1.00 (reference)	
	19	18	37	18	4.54 (2.19, 9.42)		4.48 (2.11, 9.5)	
	13	11	24	117	4.37 (1.82, 10.51)	<0.01	4.53 (1.79, 11.5)	<0.01
Total	454	131	585					
Rectum								
Men and women	1,015	161	1,176	Non-intake	1.00 (reference)		1.00 (reference)	
	50	20	70	18	2.82 (1.62, 4.91)		2.73 (1.54, 4.82)	
	31	19	50	117	4.16 (2.27, 7.63)	<0.01	4.24 (2.29, 7.84)	<0.01
Total	1,096	200	1,296					
Men	593	92	685	Non-intake	1.00 (reference)		1.00 (reference)	
	31	10	41	18	2.22 (1.04, 4.70)		2.61 (1.19, 5.74)	
	18	9	27	117	3.38 (1.46, 7.79)	<0.01	3.49 (1.48, 8.23)	<0.01
Total	642	111	753					
Women	422	69	491	Non-intake	1.00 (reference)		1.00 (reference)	
	19	10	29	18	3.84 (1.66, 8.90)		3.24 (1.35, 7.77)	
	13	10	23	117	5.35 (2.17, 13.18)	<0.01	5.50 (2.10, 14.39)	<0.01
Total	454	89	543					

PhIP intake (median, ng/day): 2-amino-1-methyl-6-phenylimidazo pyridine (PhIP); ^a^ CRC, colorectal cancers;^ b^ Adjusted for age groups (0-29, 30-39, 40-49, 50-59, 60-69, ≥70 ages) for men and women; and age groups and sex for the total; c Multivariable adjusted for age groups (0-29, 30-39, 40-49, 50-59, 60-69, ≥70 ages) for men and women; and age groups and sex for total; BMI (18.5 to <23, 23 to <25, ≥25, <18.5 kg/m^2^); education level (Primary school or under, secondary school, high school, higher high school, unknown); Frequency of intake of vegetables (Water spinach, mustard greens, sauropus, Malabar nightshade, cabbage); and lifetime smoking (yes/no)

**Table 2:** PhIP from Beef Intake and Risk of Sub-Sites of Proximal and Distal Colon Cancer

	Control	Cancer	Total	PhIP intake (Median)	Adjusted ^a^ OR (95%CI)	*P_trend*	Multivariable adjusted^b ^OR (95%CI)	*P_trend*
Proximal colon								
Men and women	1,015	75	1,090	Non-intake	1.00 (reference)		1.00 (reference)	
	50	13	63	18	4.10 (2.08, 8.08)		3.91 (1.95, 7.83)	
	31	14	45	97.5	8.56 (4.17, 17.55)	<0.01	8.05 (3.86, 16.77)	<0.01
Total	1,096	102	1,198					
Men	593	45	638	Non-intake	1.00 (reference)		1.00 (reference)	
	31	8	39	18	3.93 (1.67, 9.23)		4.05 (1.66, 9.89)	
	18	8	26	87	7.81 (3.07, 19.9)	<0.01	6.91 (2.65, 18.07)	<0.01
Total	642	61	703					
Women	422	30	452	Non-intake	1.00 (reference)		1.00 (reference)	
	19	5	24	18	4.24 (1.39, 12.95)		4.33 (1.33, 14.03)	
	13	6	19	117	9.97 (3.24, 30.64)	<0.01	12.15 (3.73, 39.57)	<0.01
Total	454	41	495					
Distal colon								
Men and women	1,015	77	1,092	Non-intake	1.00 (reference)		1.00 (reference)	
	50	13	63	18	4.09 (2.09, 8.02)		3.34 (1.66, 6.73)	
	31	3	34	117	1.57 (0.46, 5.34)	<0.01	1.69 (0.49, 5.86)	0.01
Total	1,096	93	1,189					
Men	593	38	631	Non-intake	1.00 (reference)		1.00 (reference)	
	31	6	37	18	3.52 (1.36, 9.13)		2.28 (0.77, 6.76)	
	18	3	21	117	3.07 (0.85, 11.15)	0.01	2.82 (0.75, 10.52)	0.05
Total	642	47	689					
Women	422	39	461	Non-intake	1.00 (reference)		1.00 (reference)	
	32	7	39	25.5	2.87 (1.15, 7.12)	0.02	3.16 (1.22, 8.17)	0.02
Total	454	46	500					

PhIP intake (median, ng/day): 2-amino-1-methyl-6-phenylimidazo pyridine (PhIP); ^a^Adjusted for age groups (0-29, 30-39, 40-49, 50-59, 60-69, ≥70 ages) for men and women; and age groups and sex for the total; ^b^Multivariable adjusted for age groups (0-29, 30-39, 40-49, 50-59, 60-69, ≥70 ages) for men and women; and age groups and sex for total; BMI (18.5 to <23, 23 to <25, ≥25, <18.5 kg/m2); education level (Primary school or under, secondary school, high school, higher high school, unknown); Frequency of intake of vegetables (Water spinach, mustard greens, sauropus, Malabar nightshade, cabbage); and lifetime smoking (yes/no)

## Results

There was 30.84% of 642 controls in men and 99.34% of 454 in women to be never lifetime smokers. Among CRC cancer, there was 71.23% of 292 men and 1.82% of 220 women categorized as ever lifetime smokers. The proportion of overweight or obese in the controls was 13.40% in men and 11.45 in women. The occurrence of CRC before the age of 30 was 1.37% in men (4 patients) and 1.36% in women (3 patients). The average age was 59.40 (25-99) in CRC patients and 54.74 (17-92) in the controls (data not shown). 

Among 1,608 participants, number beefeaters of cooked-beef (i.e. pan-fried hamburger, pan-fried steak, roast beef, and barbecued steak) were 218 (12.5%). When men and women combined, the median intake of PhIP from beef per person per day was 18ng and 102ng for the medium and high amount of intake per day, respectively.

Overall the multivariable adjusted risk significantly increased due to a high intake of PhIP from beef: OR (95%CI): 4.89 (3.03, 7.89), *p_trend*<0.01 for total participants, OR (95%CI): 5.27 (2.83, 9.81), *p_trend*<0.01 for men, and OR (95%CI): 4.58 (2.10, 10.01), *p_trend*<0.01 for women. A similar observation was seen for colon OR (95%CI): 5.68 (3.31, 9.76), *p_trend*<0.01 for total colon cancer, OR (95%CI): 6.67 (3.35, 13.3), *p_trend*<0.01 for men, and OR (95%CI): 4.53 (1.79, 11.5), *p_trend*<0.01 for women. For rectum, OR (95%CI): 4.24 (2.29, 7.84), *p_trend*<0.01 for total rectum cancer, OR (95%CI): 3.49 (1.48, 8.23), *p_trend*<0.01 for men, and OR (95%CI): 5.50 (2.10, 14.39), *p_trend*<0.01 for women, Table 1.

By subsides, the significantly increased risk was observed for proximal colon, OR (95%CI): 8.05 (3.86, 16.77), *p_trend*<0.01 for total proximal colon, OR (95%CI): 6.91 (2.65, 18.07), *p_trend*<0.01 for men, and OR (95%CI): 12.15 (3.73, 39.57), *p_trend*<0.01 for women. For distal colon, OR (95%CI): 1.69 (0.49, 5.86), *p_trend*<0.01 for total distal colon, OR (95%CI): 2.82 (0.75, 10.52), *p_trend*=0.05, for men, Table 2.

By smoking status, the elevated risk was seen for the never-lifestyle smokers, OR (95%CI): 4.78 (2.55, 8.95), *p_trend*<0.01, men and women combined, OR (95%CI): 5.07 (1.76, 14.61), *p_trend*<0.01 for men, and OR (95%CI): 4.40 (1.99, 9.70), *p_trend*<0.01 for women. For ever-lifestyle smokers, OR (95%CI): 5.58 (2.55, 12.20), *p_trend*<0.01, men and women combined, OR (95%CI): 5.37 (2.44, 11.84), *p_trend*<0.01 for men. Data was not available for women due to a small number of women smokers, (data not shown). 

## Discussions

An unhealthy diet continues to be a major public health issue worldwide, especially in low- and middle-income countries, including Viet Nam. Also, the underlying etiology of an unhealthy diet induces cancer and non-communicable diseases remain unclear to date. The present case-control study shows that PhIP from beef-related to the significantly increased risk of CRC in the beefeaters when compared to the reference group of non-beefeaters. This is the most comprehensive and up-to-date observational study in Viet Nam and the Asia region. We observed a strong positive association between intakes of PhIP from beef and the development of CRC in total and their sub-site of the colon (included proximal and distal colon) and rectum cancer in both men and women. The role of PhIP from beef-related to CRC cancer appears to be independent with tobacco smoking when a strong positive association between intakes of PhIP from beef and the development of CRC remains statistically significant in the sub-group of never lifetime smokers in both men and women. Our findings support to the hypotheses that the group of heterocyclic amines occurred in cooked meat, especially in cooked beef (i.e. pan-fried hamburger, pan-fried steak, roast beef, and barbecued steak) is possibly carcinogenic to humans (Group 2B) (IARC, 1993; NTP, 2014; Sugimura, 1997). Our study demonstrated that there is a significantly increased risk of CRC in both men and women, both proximal and distal colon in our study population. Innovative strategies in promoting a healthy diet to reduce consuming red meat included beef will likely be needed to prevent the occurrence of CRC and its sub-sites. 

The National Toxicology Program. Department of Health and Human Services concluded: “PhIP is reasonably anticipated to be a human carcinogen based on sufficient evidence of carcinogenicity from studies in experimental animals and supporting nontoxicity data” (IARC, 1993; NTP, 2014). Heterocyclic amines included PhIP are generated when meats are cooked at high cooking temperatures resulted from the reaction between amino acids, sugars, and creatinine (Hodge, 1953; Maillard, 1912). Over 20 types of heterocyclic amines have been identified in cooked meats, and concentrations appear to increase with higher temperatures and longer duration of cooking (Sugimura, 1997; Turesky et al., 2007). 

The strengths of the present study include a large sample size of 512 CRC cases and 1,096 non-cancer controls. We used local analyzed data of PhIP concentration in cooked beef by the LC/MS/MS method that will reflect a real current intake of PhIP in our study population. Using the validated SQFFQ, demographic, and lifestyle cooking method questionnaires, we obtained data of food frequencies of intake of cooked beef and other foods/recipes with reasonable completeness and reliable quality to estimate the amount of PhIP intake by participants. With these advanced approaches, we explored the positive association between heterocyclic amines of PhIP from beef and risk of CRC for the entire study population, in both men and women, for sub-types of the proximal and distal colon, and both never- and ever-lifetime tobacco smokers. Our findings are consistent with the previous results confirmed the positive association between heterocyclic amines intake and the risk of CRC or its sub-sites(Butler et al., 2003; Helmus et al., 2013; Joshi et al., 2015; Le Marchand et al., 2002; Miller et al., 2013; Nowell et al., 2002). The present findings were consistent with the previous study on the association between cooking method and cancer risk in the North Viet Nam. A high intake of roasted meats including beef significantly increased the risk of stomach and colorectal cancers (Ngoan et al., 2009). 

A few studies have examined the relationship between heterocyclic amines included PhIP and risk of CRC, and the results are inconsistent. Some U.S. based case-control studies on HCAs and CRC observed a positive association between HCA intake and CRC or colon cancer (Butler et al., 2003; Helmus et al., 2013; Joshi et al., 2015; Le Marchand et al., 2002; Miller et al., 2013; Nowell et al., 2002). A null association between HCA intake and CRC or colon cancer was observed in the other studies (Augustsson et al., 1999; Gilsing et al., 2012; Kobayashi et al., 2009). Two large prospective cohort studies in the U.S. have previously reported the association between HCAs and the risk of CRC. However, the positive association between HCAs and the risk of CRC was seen on the NIH-AARP study (Cross et al., 2010) but not in the Multiethnic Cohort study (Ollberding et al., 2012). Differences in exposure assessment using cooking questionnaires and the use of the different HCA database may at least partly explain those differential results. 

Our study has some limitations. This case-control study recalled exposure data and information that is a limitation of methodological approaches. Data on PhIP intake is just available for cooked-beef, while PhIP concentration identified in many other cooked types of meat, chicken, fishes (IARC, 1993; NTP, 2014; Sugimura, 1997). In spite of these limitations, the present results warrant further investigation for heterocyclic amines, meat-derived mutagenicity (MDM), 2-amino-3,8-dimethylimidazo(4,5-j)quinoxaline (MeIQx), 2-amino-1-methyl-6-phenylimidazo(4,5-b)pyridine (PhIP), 2-amino-3,4,8-trimethylimidazo(4,5-f)quinoxaline (DiMeIQx). For distal colon cancer, in men, only 58 participants were included in the final analytical model, and the positive association was at the borderline of statistical analysis with *p_trend*=0.05, due to the relatively small number of cancer for this sub-site.

In conclusion, we observed the positive association of PhIP from beef intake and the development of CRC. Cooking methods related to the PhIP occurrence in beef recipes would be a significant investigation for our healthy diet against CRC in particular and non-communicable diseases in general. 

## References

[B1] Augustsson K, Skog K, Jagerstad M, Dickman PW, Steineck G (1999). Dietary heterocyclic amines and cancer of the colon, rectum, bladder, and kidney: a population-based study. Lancet.

[B2] Butler LM, Sinha R, Millikan RC (2003). Heterocyclic amines, meat intake, and association with colon cancer in a population-based study. Am J Epidemiol.

[B3] Cross AJ, Ferrucci LM, Risch A (2010). A large prospective study of meat consumption and colorectal cancer risk: an investigation of potential mechanisms underlying this association. Cancer Res.

[B4] Gilsing AM, Berndt SI, Ruder EH (2012). Meat-related mutagen exposure, xenobiotic metabolizing gene polymorphisms, and the risk of advanced colorectal adenoma and cancer. Carcinogenesis.

[B5] Helmus DS, Thompson CL, Zelenskiy S, Tucker TC, Li L (2013). Red meat-derived heterocyclic amines increase the risk of colon cancer: a population-based case-control study. Nutr Cancer.

[B6] Hodge JE (1953). Dehydrated foods, the chemistry of browning reactions in model systems. J Agric Food Chem.

[B9] Joshi AD, Kim A, Lewinger JP (2015). Meat intake, cooking methods, dietary carcinogens, and colorectal cancer risk: findings from the Colorectal Cancer Family Registry. Cancer Med.

[B10] Kobayashi M, Otani T, Iwasaki M (2009). Association between dietary heterocyclic amine levels, genetic polymorphisms of NAT2, CYP1A1, and CYP1A2 and risk of colorectal cancer: a hospital-based case-control study in Japan. Scand J Gastroenterol.

[B11] Le Marchand L, Hankin JH, Pierce LM (2002). Well-done red meat, metabolic phenotypes, and colorectal cancer in Hawaii. Mutat Res.

[B12] Le TN, Le XH, Pham VP (2018). Reproducibility of a designed semi-quantitative food frequency questionnaire in general populations in the North Viet Nam. Southeast Asian J Sci.

[B14] Maillard LC (1912). Action of amino acids on sugars Formation of melanoidins in a methodical way. Compt Rend.

[B15] Miller PE, Lazarus P, Lesko SM (2013). Meat-related compounds and colorectal cancer risk by anatomical subsite. Nutr Cancer.

[B17] Ngoan LT, Thu NT, Lua NT (2009). Cooking temperature, heat-generated carcinogens, and the risk of stomach and colorectal cancers. Asian Pac J Cancer Prev.

[B18] Nowell S, Coles B, Sinha R (2002). Analysis of total meat intake and exposure to individual heterocyclic amines in a case-control study of colorectal cancer: contribution of metabolic variation to risk. Mutat Res.

[B20] Ollberding NJ, Wilkens LR, Henderson BE, Kolonel LN, Le Marchand L (2012). Meat consumption, heterocyclic amines, and colorectal cancer risk: the Multiethnic Cohort Study. Int J Cancer.

[B21] Sinha R, Rothman N, Salmon CP (1998). Heterocyclic amine content in beef cooked by different methods to varying degrees of doneness and gravy made from meat drippings. Food Chem Toxicol.

[B22] Sugimura T (1997). Overview of carcinogenic heterocyclic amines. Mutat Res.

[B23] Thuong NV 2018.

[B24] Turesky RJ, Goodenough AK, Ni W (2007). Identification of 2-amino-1,7-dimethylimidazo[4,5-g]quinoxaline: an abundant mutagenic heterocyclic aromatic amine formed in cooked beef. Chem Res Toxicol.

